# Mapping of quantitative trait loci for flesh colour and growth traits in Atlantic salmon (*Salmo salar*)

**DOI:** 10.1186/1297-9686-42-17

**Published:** 2010-06-04

**Authors:** Matthew Baranski, Thomas Moen, Dag Inge Våge

**Affiliations:** 1 Nofima Marin, P.O. Box 5010, 1432 Ås, Norway; 2 Department of Animal and Aquacultural Sciences, Norwegian University of Life Sciences, P.O. Box 5003, 1432 Ås, Norway; 3 The Centre for Integrative Genetics (CIGENE), Norwegian University of Life Sciences, P.O. Box 5003, 1432 Ås, Norway; 4 Aqua Gen AS, Postboks 1240, Pirsenteret, 7462 Trondheim, Norway

## Abstract

**Background:**

Flesh colour and growth related traits in salmonids are both commercially important and of great interest from a physiological and evolutionary perspective. The aim of this study was to identify quantitative trait loci (QTL) affecting flesh colour and growth related traits in an F2 population derived from an isolated, landlocked wild population in Norway (Byglands Bleke) and a commercial production population.

**Methods:**

One hundred and twenty-eight informative microsatellite loci distributed across all 29 linkage groups in Atlantic salmon were genotyped in individuals from four F2 families that were selected from the ends of the flesh colour distribution. Genotyping of 23 additional loci and two additional families was performed on a number of linkage groups harbouring putative QTL. QTL analysis was performed using a line-cross model assuming fixation of alternate QTL alleles and a half-sib model with no assumptions about the number and frequency of QTL alleles in the founder populations.

**Results:**

A moderate to strong phenotypic correlation was found between colour, length and weight traits. In total, 13 genome-wide significant QTL were detected for all traits using the line-cross model, including three genome-wide significant QTL for flesh colour (Chr 6, Chr 26 and Chr 4). In addition, 32 suggestive QTL were detected (chromosome-wide P < 0.05). Using the half-sib model, six genome-wide significant QTL were detected for all traits, including two for flesh colour (Chr 26 and Chr 4) and 41 suggestive QTL were detected (chromosome-wide P < 0.05). Based on the half-sib analysis, these two genome-wide significant QTL for flesh colour explained 24% of the phenotypic variance for this trait.

**Conclusions:**

A large number of significant and suggestive QTL for flesh colour and growth traits were found in an F2 population of Atlantic salmon. Chr 26 and Chr 4 presented the strongest evidence for significant QTL affecting flesh colour, while Chr 10, Chr 5, and Chr 4 presented the strongest evidence for significant QTL affecting growth traits (length and weight). These QTL could be strong candidates for use in marker-assisted selection and provide a starting point for further characterisation of the genetic components underlying flesh colour and growth.

## Background

Carotenoid uptake and subsequent deposition in the muscle of fish such as salmon, trout and char is a heritable quantitative trait that is commercially very important for the aquaculture industry [1-3]. Astaxanthin is an expensive ingredient in fish feed (5-10% of feed cost) and muscle deposition of colour in the fish is relatively poor [4,5]. Market preference for red-fleshed fish has made flesh colour an important trait for breeding goals in Atlantic salmon selection programs. However, at present flesh colour cannot be accurately measured on live adult individuals. Consequently, no within-family selection can be performed and only part of the genetic variation of the trait can be exploited. Marker assisted selection (MAS) using markers linked to quantitative trait loci (QTL) for flesh colour represents an excellent way to improve the efficiency of selection. Heritabilities for flesh colour in Atlantic salmon tend to be low when subjective colour card measurements are used and medium when measurements are based on instrumental methods, with a reported range generally between 0.1 and 0.2 [6,7].

The extent of genetic control of pigmentation in salmonids has not been conclusively demonstrated. A cross between extremely strong- and weak-coloured populations of Chinook salmon exhibited a phenotypic distribution originally explained by a model involving two loci, each with two alleles [8]. The proposed model could not explain the anomalous red:white ratios among the progeny of one male parent. A recent study has shown that this dataset could be fully explained by a model with one locus and three alleles [9]. In another study [6], a single locus SCAR marker with a relatively strong association to flesh colour in Coho salmon has been identified, suggesting that the genetic control of flesh colour may be controlled by relatively few loci with large effects, rather than a large polygenic effect. A dynamic model of carotenoid metabolism in salmonids, based on ordinary differential equations, has identified the uptake process of carotenoid over the muscle membrane as a potential important source of genetic variation [10]. Given that this model mimics the real situation, the existence of key regulatory sites could possibly suggest the presence of loci with relatively large effects. However this does not necessarily mean that the trait will be regulated via polymorphisms with major effects within the genes encoding these sites.

An F2 population is a useful design to detect loci affecting QTL when two phenotypically distinct populations are crossed [11]. In Atlantic salmon, such populations are relatively rare, and the production of divergent or inbred lines is a long term undertaking due to the long generation interval. However, isolated populations of Atlantic salmon do exist in Norway, and show clear evidence of substantial phenotypic differences from production fish that have been under artificial selection for several generations. The Bleke salmon is a freshwater Atlantic salmon population inhabiting the inner part of the Byglandsfjord in southern Norway. This slow-growing ice age relict was isolated from sea-migrating populations about 9000 years ago because of a waterfall barrier (Vigelandsfoss) [12]. Female Bleke salmon become sexually mature after 4-5 years of freshwater life at a size of about 25 cm fork length [13] compared to that of 70-120 cm in ancestral migratory populations. In 1999, Bleke salmon were crossed to commercial Norwegian salmon selected for fast growth and high colour. The resulting F1 were then crossed to produce an F2 mapping population suitable for the detection of QTL for flesh colour, growth rate and other traits diverging between the parental populations. The aim of our study was to identify QTL affecting flesh colour and growth traits in this F2 population.

## Methods

### Mapping population

**Figure 1:**
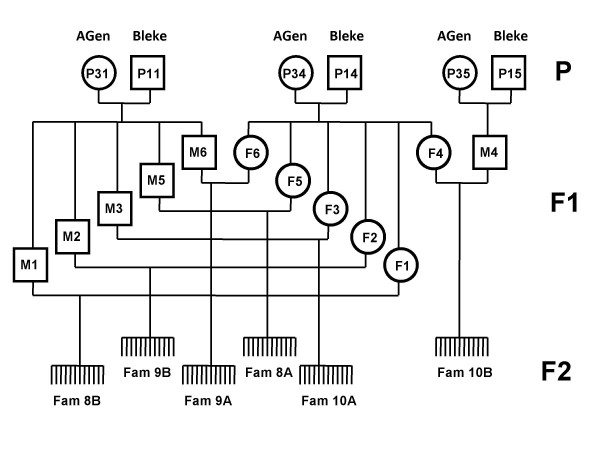
**Pedigree of the mapping population**. Founding generation (P) consisting of Bleke males (Bleke) and Aqua Gen females (AGen).

The mapping population consisted of six F2 families that originated from a cross between two divergent populations, the landlocked Byglands Bleke population and a commercial breeding population under selection (Aqua Gen AS). In 1995, three Bleke salmon were crossed with three commercial Norwegian salmon, forming three full-sib families. Five F1 males from one family and five F1 females from another family were subsequently crossed to produce five full-sib F2 families, in addition to a sixth F2 family that was sired by a male from the third F1 family. The pedigree is depicted in Figure 1.

### Phenotypic data

F2 progeny were slaughtered at three years of age and had the following traits recorded: length (L), body weight (BW), slaughter weight (SW), and colour (C) in SalmoFan™ colour units. In addition, Fulton's condition factor (*K*), a measure of a fish's girth, was calculated as (BW × L^3 ^× 100) [14] and dressing percentage (D%) was calculated as ((BW-SW)/BW × 100). Samples that were paler than the palest colour value (20) on the SalmoFan were given the score 19. Not all the individuals had sufficient gonad developed to be sexed at sampling. For the unsexed progeny, paternal allelic segregation at the microsatellite locus Ssa202DU, known to be tightly linked to the sex-determining locus [15], was used to divide the progeny into males and females. The appropriate marker phase was established from the sexed progeny in each family.

### Genotyping

Fifty progeny from each extreme of the colour distribution were selected from three F2 families (8B, 9B and 10B), and all 76 progeny from a fourth family (10A) were selected for genotyping. Corrected values for colour based on the fish size correlation were not used in this selection in order to provide sufficient power for QTL detection for the other traits. Due to differences in progeny numbers between the families, this represented selective genotyping fractions (both extremes) of 44%, 35%, 35% and 100% respectively for families 8B, 9B, 10B and 10A (Table 1). Following the initial QTL analysis, 384 additional individuals were selected from the remaining extremes of the colour distribution from families (8B, 9B and 10B) as well as 384 individuals from two additional families (8A and 9A) for subsequent genotyping at putative QTL.

**Table 1:** Number of F2 progeny in each family and selective genotyping fractions

Family	Total indiv.	Sel 1 (SG%)^1^	Sel 1+2 (SG%)^2^
8A	300	-	252 (84)
8B	228	100 (44)	221 (97)
9A	157	-	132 (84)
9B	287	100 (35)	232 (81)
10A	76	76 (100)	76 (100)
10B	286	100 (35)	225 (79)

^1 ^Number of animals selected from each family for initial genome scan (selective genotyping percentage across both tails)

^2 ^Number of animals selected from each family after extra animals were added in the second round of genotyping (selective genotyping percentage across both tails)

DNA extraction was carried out from muscle tissue samples using the DNeasy 96 kit (QIAGEN) following the manufacturer's protocol. The majority of microsatellite markers used in this study were chosen from the SALMAP microsatellite map of Atlantic salmon [16], covering all 29 linkage groups (chromosomes). The nomenclature of chromosomes follows that introduced by Philips et al. [17]. In total, 128 informative microsatellite loci were initially genotyped, including duplicated loci amplified from the same primer pair (see additional file 1 for names and female map positions). Following the initial analysis, 23 additional loci were genotyped. The microsatellite markers were distributed across 32 PCR multiplexes that were subsequently combined into 16 multiplexes for capillary electrophoresis. Primer sequences and multiplex information are available on request. Polymerase chain reactions (PCR) were performed in volumes of 5 μL, using 0.25 units of AmpliTaq Gold (Applied Biosystems), 250 μM dNTP mix, 1.5 mM MgCl_2_, 0.25-1 pmol of each primer (depending on amplification efficiency of each marker in multiplex), 0.25 μL DMSO, and 5 ng DNA template. PCR cycling conditions were 95°C for 10 min, 35 cycles at 94°C for 30 seconds, 54°C for 1 min, and 72°C for 1 min, followed by a final extension at 60°C for 45 min. The lengths of the fluorescent PCR products were determined relative to the LIZ500 size standard (Applied Biosystems) on a 3730 DNA Analyzer (Applied Biosystems), using GeneMapper 4.0 (Applied Biosystems) software for allele calls.

### Construction of linkage map

Since samples of the F1 parents were not available, genotypes had to be inferred from the grandparent and progeny genotypes. A custom Visual Basic for Applications program in Excel was used for this task. In situations where it was equally likely for a parental genotype to fit the sire or dam, then, the genotype was arbitrarily applied, the linkage relationship to adjacent markers examined, and finally the parental genotypes reversed if necessary (i.e. if the marker was not linked when it should have been). Separate male and female maps were constructed due to large sex-specific recombination differences observed in salmonids [18]. Marker grouping and initial marker ordering was done with Joinmap 3.0 [19]. A Joinmap input file was made for each mapping parent (in double haploid format), containing information on alleles inherited from that parent only. Marker grouping was performed at a minimum LOD score of 4.0. Following marker grouping, homologous linkage groups from each sire and each dam were integrated into single sex-specific maps. The data was examined for unlikely double recombinants and for inconsistencies in marker order between parents using a custom VBA program in Excel (available by request from the authors). Occurrences of double recombinants over small distances were checked for genotyping errors. After marker orders and potential genotype errors had been verified, the final maps were constructed using Joinmap. The Kosambi mapping function was used.

### Interval mapping analyses

Interval mapping using regression methods was applied to two different genetic models: (1) line-cross analysis following Haley et al. [20] assuming founder lines to be fixed for different QTL alleles and (2) half-sib model [21], making no assumptions about the fixation of QTL alleles in the founder lines. In the line-cross model, QTL effects are partitioned into additive and dominance effects. The additive effect was estimated as half the difference between the phenotypic values for homozygotes for the Aqua Gen and Bleke alleles at the QTL, with a positive or a negative sign indicating that the Aqua Gen or the Bleke allele, respectively, increased the value of the trait score. The dominance effect was calculated as the phenotypic deviation of the heterozygotes from the mean of the two homozygotes. GridQTL software [22] was used for QTL analyses. Due to the significant effect of sex on the traits under study, sex was included as a fixed effect for the analysis in both models, based on records of sexed individuals and marker segregation at Ssa202DU. In the initial QTL analysis including four families, male and female mapping parents were analysed separately under the half-sib model. In the subsequent analysis with the larger data set, a joint analysis of male and female mapping parents in the half-sib model was performed by duplicating the dataset prior to analysis, with the designation of parents as sire or dams inverted in the duplicate. In the initial QTL analysis, length was included as a covariate for the analysis of colour, however in the subsequent analysis, body weight was used as the covariate. Full-sib family was fitted as a fixed effect in the line-cross model in the larger dataset (but was omitted in the initial analysis).

P values were calculated for all trait-by-chromosome combinations with the significance of the peak F-statistic (putative QTL) estimated after 10,000 chromosome-wide permutation tests [23]. The chromosomal location of the QTL was taken as the position with the highest F-statistic. Two levels of significance are reported for the detected QTL. A QTL was found to be genome-wide significant if the chromosome-wide significance level was smaller than 0.05 * 29, a Bonferroni correction based on the number of linkage groups examined. QTL that were chromosome-wide significant at P < 0.01 and P < 0.05 but not genome-wide significant were regarded as 'suggestive' QTL. Because this was an initial scan, and also for ease of comparison of the results with those of other studies (as suggested by [24]), correction for multiple traits was not performed. The proportion of phenotypic variance explained by the QTL using the half-sib model was calculated as 4*(1-MS_full_/MS_reduced_) where MS_full _is the mean squared error of the full model, accommodating one QTL effect for each informative mapping parent, while MS_reduced _is the corresponding mean squared error of the reduced model omitting QTL effects [21]. Correction for overestimation of QTL effects due to selective genotyping for flesh colour was not performed due to the different selective genotyping fractions in each family and to the fact that almost all individuals within each family were ultimately genotyped for the four linkage groups that were further investigated. In addition, this correction was not applied for the other traits due to the fact that progeny were only selected from the extremes of the colour distribution and not for these traits (however, the positive correlation between length, weight and colour will mean that some selective genotyping has taken place, and some QTL effect overestimation has occurred). Confidence intervals (CI) were estimated for each genome-wide significant QTL using the bootstrap method [25] and 10,000 iterations.

## Results

### Phenotypic data analysis

**Figure 2:**
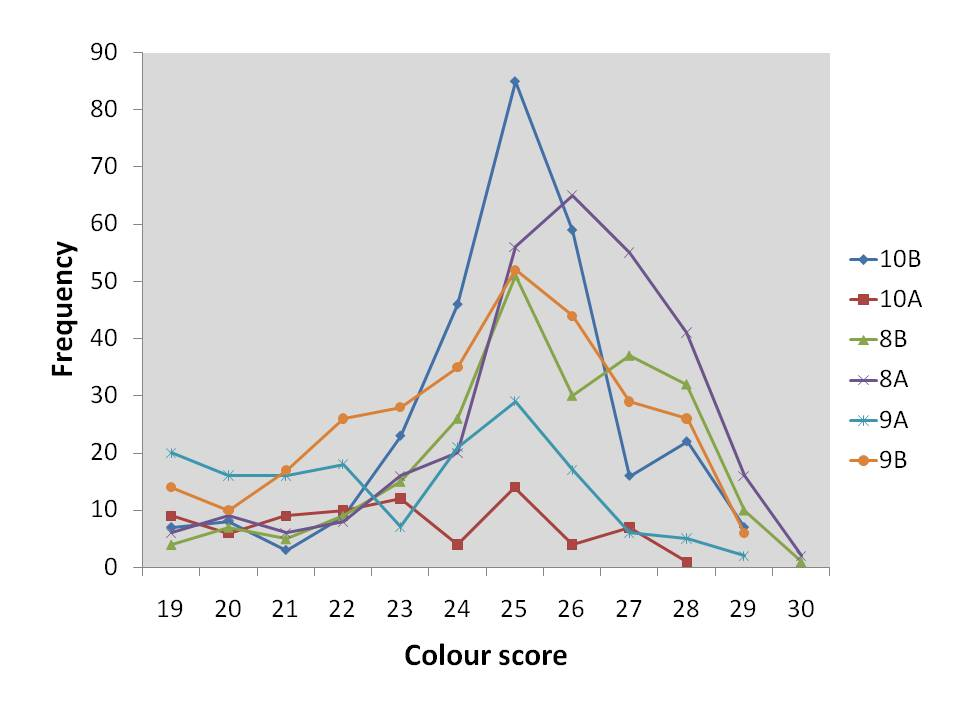
**Colour frequency distribution**. Frequency distribution of colour scores over the six F2 families.

Analysis of raw phenotypic data in the F2 population revealed that all traits exhibited substantial levels of phenotypic variation (Table 2), and strong phenotypic correlations were observed between numerous traits (Table 3). Flesh colour was moderately to strongly correlated to length (0.76), body weight (0.75) and slaughter weight (0.74). Colour was also moderately correlated to *K *factor (0.60) and weakly correlated to dressing percentage (0.20). There were significant differences in all trait averages between the two sexes (P < 0.001). A total of 6% of all F2 progeny had flesh colour scores below the minimum SalmoFan value of 20, and were therefore given the score 19 for this trait (Figure 2).

**Table 2:** Phenotypic averages of F2 families. Phenotypic averages and standard deviations (in parentheses) for traits recorded in the six F2 families

Family	L (cm)	BW (kg)	SW (g)	*K*	SL (%)	C^1^
8A	62.6 (8.4)	3.39 (1.34)	3.03 (1.21)	1.38 (0.14)	10.6 (1.6)	25.7 (2.3)
8B	60.0 (8.1)	2.95 (1.25)	2.65 (1.13)	1.36 (0.16)	10.4 (1.8)	25.4 (2.8)
9A	54.8 (11.0)	2.20 (1.44)	2.00 (1.32)	1.3 (0.24)	9.2 (2.0)	23.7 (2.7)
9B	57.6 (9.1)	2.60 (1.29)	2.32 (1.16)	1.4 (0.20)	10.7 (2.2)	24.7 (2.5)
10A	55.7 (10.6)	2.30 (1.55)	2.07 (1.39)	1.3 (0.25)	10.1 (1.8)	23.5 (2.5)
10B	59.3 (8.8)	2.96 (1.26)	2.67 (1.14)	1.4 (0.16)	10.0 (3.3)	25.0 (2.4)

^1^SalmoFan colour score units

**Table 3:** Phenotypic correlations between carcass traits. Phenotypic correlations between carcass traits

	BW	SW	K	D%	C
**L**	0.96	0.96	0.49	0.12	0.76
**BW**		1.00	0.58	0.10	0.75
**SW**			0.56	0.06	0.74
**K**				0.36	0.60
**D%**					0.20

### QTL results - Initial genome scan

An initial genome scan was performed using four of the six full-sib families, for the traits flesh colour, body weight and length. Under the across family half-sib model, genome-wide significant QTL were identified for flesh colour on Chr 4, for body weight on Chr 4 and for length on Chr 10 and Chr 4 (Table 4). All QTL were detected in the sire-based analysis. Under the line-cross model, genome-wide significant QTL were identified for flesh colour on Chr 4, for body weight on Chr 5 and Chr 4 and for length on Chr 10 and Chr 4 (Table 4). Numerous additional suggestive QTL were also detected. Genome-wide significance in either model was used as criteria to select chromosomes 10, 5, and 4 for genotyping in additional samples. In addition, suggestive evidence for a colour QTL on Chr 26 under both models was used as criteria for selection of Chr 26 for additional genotyping. Seven hundred and sixty-two additional animals were genotyped for markers on chromosomes 10, 5, 4, and 26. To improve coverage, 23 additional microsatellites were genotyped for chromosomes 26 and 4 (see Additional File 1).

**Table 4:** Initial QTL analysis using half-sib and line cross models

Half-sib model^a^			Line-cross model		
Trait	Chr	F	Trait	Chr	F
Flesh colour	4	18.15***	Flesh colour	4	12.31***
	26	3.92**		6	5.64*
	5	3.38*		5	5.3*
	1	3.13*		26	5.27*
	9	3.02*		7	5.05*
	19	2.85*		2	4.85*
	8	2.78*			
	13	2.63*			
Body weight	4	16.21***	Body weight	4	15.68***
	5	3.84**		5	7.91***
	16	3.79**		10	7.57**
	10	3.59**		7	6.64**
	13	3.21*		18	3.95*
	2	3.07*			
	7	2.92*			
	11	2.62*			
Length	4	14.41***	Length	4	17.9***
	10	4.58***		10	10.26***
	13	4.01**		5	7.91**
	16	4.01**		11	5.4**
	5	3.7**		7	4.83*
	11	3.27*		18	3.95*
	2	2.83*			
	7	2.81*			

a Sire-based analysis

*** Genome-wide significant QTL (P < 0.05)

** Chromosome-wide significant QTL (P < 0.01)

* Chromosome-wide significant QTL (P < 0.05)

### QTL results - Full dataset with the line-cross model

**Figure 3:**
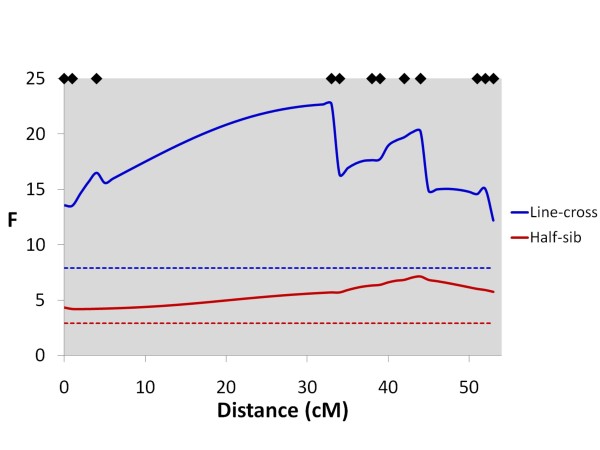
**Line-cross and half-sib interval mapping analysis for flesh colour on Chr 26**. F-statistic profiles for Chr 26 for both line-cross and half-sib models for flesh colour; diamonds on the top axis represent marker positions; horizontal dashed lines represent genome-wide significance thresholds (P < 0.05) for both line-cross (blue) and half-sib (red) analyses.

**Figure 4:**
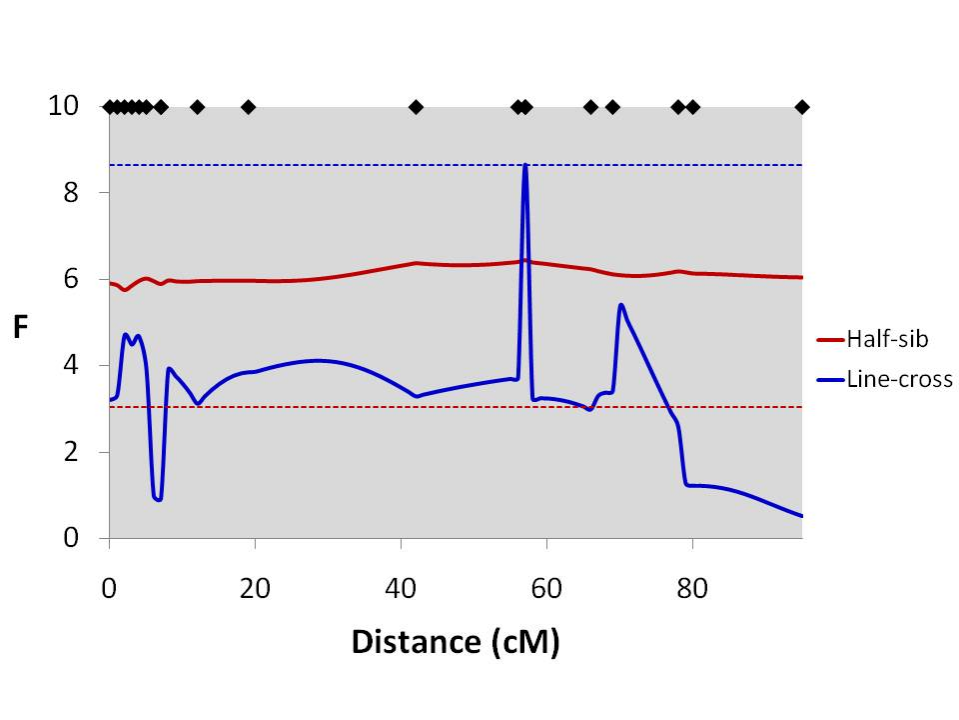
**Line-cross and half-sib interval mapping analysis for flesh colour on Chr 4**. F-statistic profiles for Chr 4 for both line-cross and half-sib models for flesh colour; diamonds on the top axis represent marker positions; horizontal dashed lines represent genome-wide significance thresholds (P < 0.05) for both line-cross (blue) and half-sib (red) analyses.

**Figure 5:**
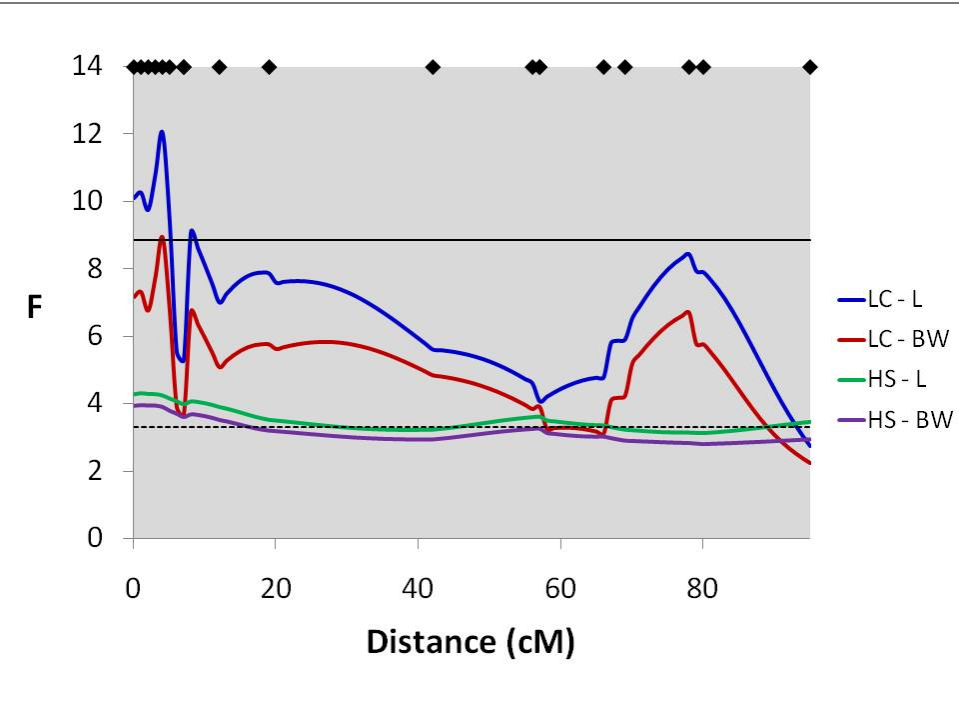
**Line-cross and half-sib interval mapping analysis for length and body weight on Chr 4**. F-statistic profiles for Chr 4 for both line-cross and half-sib models for length and body weight; diamonds on the top axis represent marker positions; horizontal solid and dashed black lines represent the genome-wide significance thresholds (P < 0.05) for both line-cross and half-sib analyses, respectively.

**Figure 6:**
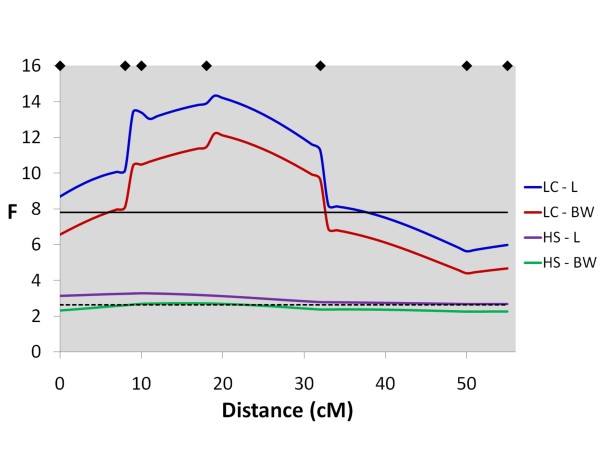
**Line-cross and half-sib interval mapping analysis for length and body weight on Chr 10**. F-statistic profiles for Chr 10 for both line-cross and half-sib models for length and body weight; diamonds on the top axis represent marker positions; horizontal solid and dashed black lines represent the genome-wide significance thresholds (P < 0.05) for both line-cross and half-sib analyses, respectively.

**Figure 7:**
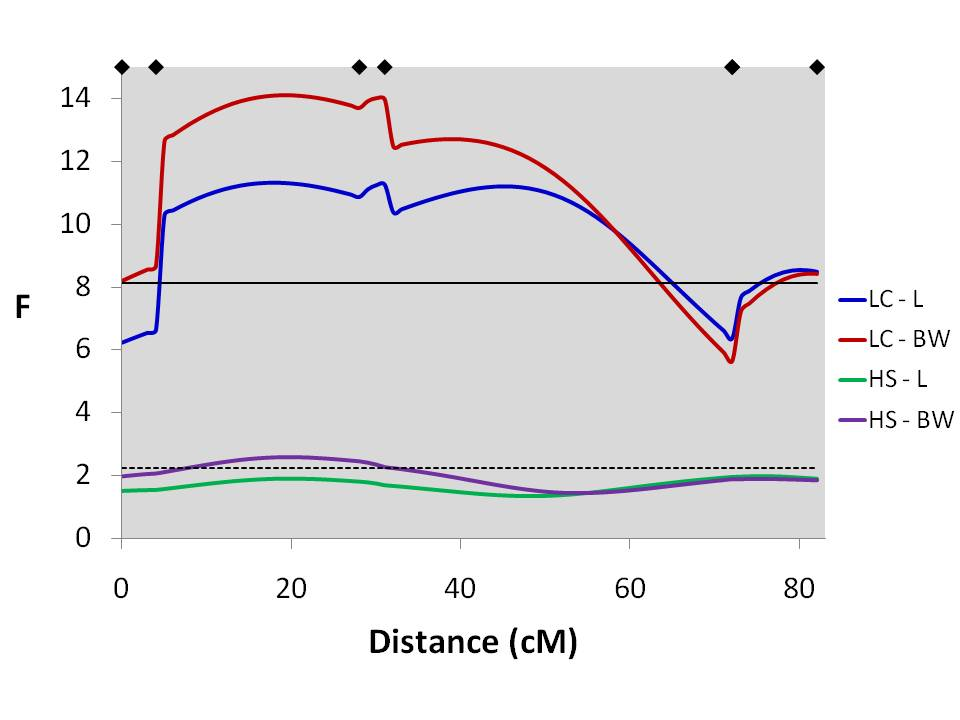
**Line-cross and half-sib interval mapping analysis for length, body weight and slaughter weight on Chr 5**. F-statistic profiles for Chr 5 for both line-cross and half-sib models for length and body weight; diamonds on the top axis represent marker positions; horizontal solid and dashed black lines represent the line-cross genome-wide significance threshold (P < 0.05) and half-sib chromosome-wide significance threshold (P < 0.05), respectively.

In total, 13 genome-wide significant QTL were detected for all traits using the line-cross model (Table 5). Five QTL were significant at the chromosome-wide P < 0.01 level, and 27 were significant at the chromosome-wide P < 0.05 level (suggestive QTL). Of the 45 significant or suggestive QTL detected, 40 had primarily additive effects, whilst five had larger dominance effects. For flesh colour, three genome-wide significant QTL were detected, two with primarily additive (Chr 26 and Chr 4) and one (Chr 6) with primarily dominance effects. Numerous linkage groups had multiple QTL mapping to them, particularly the strongly correlated length, body weight and slaughter weight traits. Genome-wide significant QTL for colour mapped uniquely to Chr 26 (Figure 3) and Chr 6, and on Chr 4 a genome-wide significant QTL peak (Figure 4) was 53 cM away from genome-wide significant QTL peaks for length and weight (Figure 5). Genome-wide significant QTL for length, body weight and slaughter weight were confirmed on Chr 10 (Figure 6) and Chr 5 (Figure 7). Based on the sign of the additive effect, only three of the 45 QTL were identified where the allele derived from the Bleke line increased the value of the trait score (positive additive effect). 95% QTL confidence intervals were large, covering nearly the entire chromosomes.

**Table 5:** Quantitative trait loci (QTL) mapped using the F2 line cross regression analysis

Trait	Chr	Pos (cM)	F-ratio	Additive effect (SE)	Dominance effect (SE)	Det HS?^a^
Flesh colour	26	33	22.73***	0.56 (0.08)	0.02 (0.14)	Y
	6	109	9.47***	-0.366 (0.151)	-0.916 (0.267)	Y^b^
	4	57	8.65***	0.279 (0.079)	-0.254 (0.124)	Y
	5	16	5.69*	0.266 (0.082)	-0.091 (0.131)	Y^b^
	20	41	5.35*	-0.428 (0.131)	0 (0.201)	Y^b^
	7	8	4.96*	0.415 (0.133)	-0.049 (0.207)	Y
	1	0	4.94*	0.04 (0.125)	-0.57 (0.185)	Y^b^
	10	18	4.92*	0.227 (0.079)	-0.156 (0.12)	
Body weight	5	19	14.09***	0.321 (0.064)	-0.132 (0.1)	Y
	10	19	12.22***	0.345 (0.07)	0.074 (0.106)	Y
	4	4	8.96***	0.26 (0.064)	0.152 (0.099)	Y
	7	4	5.83**	0.332 (0.105)	-0.155 (0.157)	Y
	18	16	4.69*	0.343 (0.128)	-0.331 (0.216)	
	29	0	4.39*	0.294 (0.102)	0.089 (0.152)	Y
	22	0	4.12*	0.266 (0.1)	-0.133 (0.142)	
	13	58	4.07*	0.15 (0.061)	-0.121 (0.091)	Y
	19	0	3.43*	0.267 (0.102)	-0.051 (0.143)	
Length	10	19	14.34***	2.545 (0.479)	0.539 (0.726)	Y
	4	4	12.05***	2.049 (0.433)	1.247 (0.673)	Y
	5	18	11.32***	1.938 (0.44)	-1.03 (0.7)	Y
	11	17	7.44***	2.204 (0.605)	0.931 (1.219)	Y
	13	59	5.12*	1.22 (0.405)	-0.554 (0.596)	Y
	19	0	4.36*	2.055 (0.696)	-0.412 (0.979)	
	2	0	4.15*	-0.994 (0.932)	4.016 (1.59)	
	7	6	4.09*	1.824 (0.727)	-1.293 (1.116)	Y
	29	0	4.07*	1.911 (0.694)	0.739 (1.036)	
	22	0	3.5*	1.622 (0.681)	-1.013 (0.973)	
Slaughter weight	5	19	13.56***	0.285 (0.058)	-0.116 (0.091)	Y
	10	19	12.24***	0.311 (0.063)	0.069 (0.096)	Y
	4	4	9.36***	0.241 (0.057)	0.137 (0.089)	Y
	7	4	5.92**	0.303 (0.095)	-0.135 (0.142)	Y
	18	16	4.67*	0.313 (0.116)	-0.285 (0.195)	
	13	59	4.43*	0.151 (0.054)	-0.066 (0.079)	
	29	0	4.37*	0.265 (0.092)	0.079 (0.137)	
	22	0	4.09*	0.242 (0.09)	-0.108 (0.128)	
K-factor	24	48	6.69**	0.044 (0.016)	0.068 (0.028)	
	20	52	6.86**	-0.052 (0.014)	0.003 (0.02)	Y
	7	8	6.12**	0.052 (0.015)	0.021 (0.023)	Y
	5	31	6.15*	0.026 (0.008)	-0.017 (0.012)	
	10	19	5.14*	0.025 (0.01)	-0.029 (0.015)	
	23	20	4.81*	0.006 (0.015)	0.068 (0.022)	
	19	0	3.72*	0.037 (0.014)	-0.025 (0.02)	
Dressing %	17	2	5.89*	-0.537 (0.173)	-0.412 (0.243)	
	13	58	4.71*	-0.262 (0.101)	-0.272 (0.152)	

*** Genome-wide significant QTL (P < 0.05)

** Chromosome-wide significant QTL (P < 0.01)

* Chromosome-wide significant QTL (P < 0.05)

^a ^Detected using the half-sib analysis

^b ^QTL peak more than 20 cM from QTL peak in half-sib analysis

### QTL results - Full dataset with the half-sib model

In total, six genome-wide significant QTL were detected for all traits using the half-sib model (Table 6). Of the 41 suggestive QTL identified, 16 QTL were significant at the chromosome-wide P < 0.01 level, and 25 were significant at the P < 0.05 level. Like the line-cross model, numerous linkage groups had multiple QTL mapping to them, with relatively conserved positions for the strongly phenotypically correlated traits. A genome-wide significant QTL for flesh colour mapped to Chr 26 (Figure 3), where no QTL for other traits was detected, and on Chr 4 a genome-wide significant flesh colour QTL peak (Figure 4) was 56 cM away from QTL peaks for length and weight. Together, the two genome-wide significant QTLs for flesh colour on Chr 26 and Chr 4 explained 24% of the phenotypic variance for this trait. Genome-wide significant and suggestive QTL were also detected for length, body weight and slaughter weight on Chr 10 (Figure 6) and Chr 5 (Figure 7). The number of parents showing statistically significant evidence for QTL segregation ranged from one to six (Table 6 and Additional File 2). In most cases, 95% QTL confidence intervals covered nearly the entire chromosome, however the flesh colour QTL interval on Chr 26 was much narrower (38-47 cM).

**Table 6:** Quantitative trait loci (QTL) mapped using the half-sib regression analysis

Trait	Chr	Pos (cM)	F-ratio	Seg pars^a^	PVE^b^	Detect LC?^d^
Flesh colour	26	44	7.14***	6^c^	12.64	Y
	4	57	6.46***	4^c^	11.28	Y
	1	33	3.69**	2	5.66	Y^e^
	9	9	3.21**	2	4.67	
	5	72	2.69**	3^c^	3.56	Y^e^
	7	11	2.66**	3	3.52	Y
	20	1	2.8*	3	3.8	Y^e^
	6	82	2.71*	2	3.63	Y^e^
	3	37	2.45*	1	3.08	
	19	1	2.35*	2	2.86	
	8	0	2.32*	3	2.81	
	29	0	2.29*	2	2.73	
Body weight	4	1	3.95***	4^c^	6.17	Y
	16	62	3.85**	3	6.01	
	7	10	3.41**	4	5.09	Y
	10	15	2.72**	3^c^	3.62	Y
	13	42	2.83*	2	3.88	Y
	25	13	2.67*	1	3.53	
	5	20	2.59*	3^c^	3.35	Y
	23	22	2.58*	2	3.34	
	11	17	2.42*	3	3	
	2	42	2.34*	2	2.85	
Length	4	1	4.31***	4^c^	6.92	Y
	10	10	3.28***	3 ^c^	4.8	Y
	16	61	3.85**	3	5.99	
	13	61	3.69**	5	5.67	Y
	11	8	3.42**	2	5.11	Y
	7	20	3.02**	3	4.28	Y
	25	15	2.96*	1	4.15	
	23	13	2.77*	2	3.75	
	24	4	2.68*	2	3.56	
Slaughter weight	4	1	4.00***	4^c^	6.27	Y
	16	61	3.91**	3	6.13	
	7	10	3.43**	4	5.13	Y
	13	60	2.83*	2	3.87	Y
	10	16	2.69**	3^c^	3.58	Y
	25	14	2.74*	1	3.69	
	23	22	2.64*	2	3.48	
	5	20	2.55*	3^c^	3.26	Y
	11	19	2.44*	3	3.05	
	2	42	2.28*	2	2.72	
K-factor	20	46	3.89**	4	6.08	Y
	7	15	3.52**	3	5.31	Y
	3	37	2.92*	2	4.06	
	1	54	2.9*	2	4.02	
	16	10	2.89*	2	3.99	
	14	6	2.76*	3	3.73	
	12	9	2.68*	2	3.55	
Dressing %	11	21	2.79**	3	3.79	

*** Genome-wide significant QTL (P < 0.05)

** Chromosome-wide significant QTL (P < 0.01)

* Chromosome-wide significant QTL (P < 0.05)

^a ^Number of segregating parents

^b ^Percentage of within family variance explained by the QTL

^c ^Segregating out of 12 parents (extra families genotyped in these linkage groups)

^d ^Detected using the line-cross analysis

^e ^QTL peak more than 20 cM from QTL peak in line-cross analysis

### QTL results - Comparison of two models

All the genome-wide significant QTL mapped using the line-cross model were genome-wide or chromosome-wide significant (P < 0.01) under the half-sib model, with the exceptions of the QTL for flesh colour on Chr 6 and the QTL for length and body weight on Chr 5. Estimates for the amount of phenotypic variance explained by each QTL in the line-cross model were generally much lower than in the half-sib model: 12.6% vs. 3.7% for colour on Chr 26; 11.3% vs. 1.3% for colour on Chr 4; 6.2% vs. 1.4% for body weight on Chr 4; 4.8% vs. 2.3% for length on Chr 10. Numerous suggestive QTL were uniquely detected by both models (Tables 5 and 6).

## Discussion

This study used an F2 resource population to identify numerous significant and suggestive QTL for flesh colour, growth and body composition traits in Atlantic salmon. Using line-cross and half-sib regression analyses, genome-wide significant QTL for flesh colour were detected on Chr 6, Chr 26 and Chr 4. Assuming a heritability between 0.1 and 0.2 [6,7,26], these QTL could underlie a large portion of the genetic variance for the trait. Salmonids with access to astaxanthin containing diets accumulate carotenoids as they grow, and this accumulation in muscle continues till the fish approach sexual maturity [27]. The ratio of absorbed to non-absorbed carotenoid increases as the fish grows, and as a result, the concentration of fillet astaxanthin normally increases with increasing fish size, which is consistent with the strong positive correlation between fish size and flesh colour observed in this study. Consequently, a large proportion of the observed variance in flesh colour can be explained by body size, reducing the power of QTL detection for this trait. Despite this, highly significant QTL were detected for flesh colour after the inclusion of body weight as a covariate, indicating that there is measurable genetic variation present in this population. Relatively few QTL studies have been carried out on flesh colour traits in salmonids. Araneda et al. [6] identified a dominant SCAR marker associated with colour in Coho salmon (*Oncorynchus kisutch*), and Houston et al. [28] found suggestive evidence for QTL in Atlantic salmon on chromosomes 16, 18 and 23. None of these QTL reached significance in our study, although chromosomes 18 and 23 reached near chromosome-wide significance. Given the relatively low number of independent loci identified in these studies, and the small number of genome-wide significant QTL found in our study, genetic control of flesh colour in salmonids may be explained by relatively few loci of large effect. However, further validation of the suggestive QTL may reveal that they contribute to a more polygenic effect.

Dahl [12] has reported that the juveniles of the Bleke strain remain in the rivers for two to four years until they reach a length of 12 cm, before migration into the Byglandsfjord, an oligotrophic lake with a poor invertebrate population and no forage fish. In the lake, the Bleke strain exhibits enhanced growth rates, while the maximum fish size generally does not exceed 30 cm and 250 g [12]. After having been landlocked for thousands of years, an adaptation to the poor growing conditions may explain the differences in growth observed between the Bleke and wild fish from the Vosso river. However, the Bleke strain exhibits enhanced growth when transferred to lakes with ample forage fish available [29]. This may suggest that environment rather than genetic effect is more responsible for poor growth. Indeed, ecological factors related to energetics and feeding are almost certainly largely responsible for the establishment of dwarfism in the population, as was documented for Lake Whitefish populations [30]. If this is the case, it represents an important deviation from the assumptions of an F2 population derived from different lines, which are typically under strong selection for particular traits (e.g. [31]). In addition, the trait variance observed in the F2 population, while large (CV = 48.2%, 16% and 15.7% for body weight, total length and K-factor respectively), was of comparable magnitude to other salmon mapping families (45.5%, 17.8% and 9.7% for the same traits) [32] and to outbred full-sib families in other species such as barramundi (*Lates calcarifer*) (CV = 45.9%, 16.4% and 8.1% for the same traits) [33].

In this study, genome-wide significant QTL for growth and body form traits were found on Chr 10 (BW, L, SW), Chr 5 (BW, L, SW) and Chr 4 (BW, L, SW). Other studies have found evidence for QTL on Chr 4 [32,34], and QTL have been reported in Arctic charr on linkage groups homologous to Chr 4 and Chr 5 [35]. In addition, numerous linkage groups harbouring suggestive QTL for body weight, length and K-factor were replicated from previous studies. Nevertheless, the large number of different QTL reported for growth traits in Atlantic salmon, in particular body weight, suggests that these traits are highly polygenic (Table 7). Another possible explanation for the different QTL reported for these traits is that different QTL may be segregating in the European and North American populations used in these studies. European and North American Atlantic salmon have been shown to be quite distinct from one another, with *F*_ST _estimates of 0.27 using microsatellites [36,37] and 0.33 using allozymes (reviewed in [38]). Therefore it is quite likely that some QTL, such as those affecting body weight, segregate in one subgroup and not in the other.

**Table 7:** Summary of significant or suggestive body weight QTL in Atlantic salmon reported from this study and the literature

Chr	This study	**Reid et al. **[34]	**Boulding et al. **[32]	**Houston et al. **[28]
1		X		
2	X	X	X	X
3		X		X
4	X	X	X	
5	X			
6				X
7	X		X	
8				X
9		X		
10	X			
11	X		X	
12		X		
13	X			X
14			X	
15		X		
16	X			
17			X	
18	X			
20				
21		X		X
22	X			
23	X		X	
24				
25	X			
26			X	
27				
28				
29	X			

The detection of QTL for multiple traits on the same linkage groups (e.g. Chr 4) can be explained by either the linkage of two QTL (one for each trait), or the presence of a single QTL with pleiotropic effects. Reid et al. [34] detected QTL for both body weight and condition factor on five linkage groups in Atlantic salmon, and argued that they may represent different sets of genes due to low genetic correlations reported between the two traits previously. For the colour and 'growth' QTL detected on Chr 4 in this study, there is evidence to suggest that these are two separate QTL, given that the QTL peaks for colour and weight are some distance apart. However, the large, overlapping confidence intervals covering these QTL in both the line-cross and half-sib models means that further analyses will be needed to confirm this. Studies on genetic correlations between flesh colour and growth have been somewhat inconclusive in salmonids. Withler and Beacham [39] have found a moderately positive genetic correlation between final body weight and flesh colour in Coho salmon, however it was not significantly different from zero (0.44 ± 0.48). Other studies have reported stronger evidence for positive genetic correlations between growth and colour in salmonids [2,40], indicating that the same sets of genes may be involved. An extremely large QTL for IPN resistance explaining nearly all the genetic variance for this trait has been identified on Chr 26 in Atlantic salmon [41], mapping to a similar position to the flesh colour QTL in this study. Although there is little published evidence for a strong genetic correlation between flesh colour and IPN resistance, genotypes at the IPN QTL have been found to be positively correlated to flesh colour (T. Moen, pers. comm.). This suggests the possibility that extreme colour phenotypes represent individuals with alternate IPN QTL alleles due to an undocumented secondary effect of IPN infection on flesh colour. One hypothesis is that a non-lethal infection of a population with IPN could result in the more resistant fish processing or depositing pigment differently to the susceptible fish, resulting in downstream differences in flesh colour that can be explained by the IPN QTL genotype.

Under the line-cross model, the QTL allele with a positive effect on the trait value (additive effect) almost exclusively originated from the commercial line for all traits. This is not surprising given that selection has been performed for a number of generations on growth and body composition traits in this population, while the Bleke population is a natural population subject to environmental selection influence alone. Although the genome-wide significant QTL were generally detected in both the line-cross and half-sib models, a large number of suggestive QTL were uniquely detected by each model. This is likely due to the underlying assumptions of the models. Mapping of QTL using F2 populations is very powerful when the assumption of QTL allele fixation in the founding lines holds true, and is quite robust to limited deviations from this ideal situation [42]. However, when there is a very large reduction of this contrast, the power of detecting the QTL using the line-cross model is substantially reduced [42]. In the extreme case where the lines do not differ with respect to the allele frequency, then the power will be equal to zero. The half-sib model is more general, with no assumption on the number and frequency of QTL alleles in the founder populations and is almost certainly more realistic for the population in this study, since both lines are outbred. In QTL studies performed in divergent pig populations and their crosses, it has been shown that even in these selected populations there is still a considerable amount of genetic variation at loci affecting traits of interest [24]. Other studies in salmonids have also indicated high levels of variability at QTL within strains. In a QTL mapping study for temperature tolerance in Arctic charr [43], it was unexpectedly found that multiple QTL were detected in pure strain parents (Fraser River and Nauyuk Lake). It was hypothesized that, under the assumption that pure strains were almost fixed for alternate alleles, greater effects would have been detected in the male F1 hybrid parent due to segregation of QTL alleles. This was inferred because these strains descend from populations that are adapted to very different thermal regimes.

The extent of QTL variability in the founding lines in our study is also apparent since the half-sib analysis shows that the QTL segregate in only a fraction of the F1 parents. For the flesh colour QTL on Chr 26 and Chr 4, the QTL appeared to be segregating in six and four parents respectively, out of 12 parents in total. For the rest of the suggestive QTL, the number of heterozygous parents ranged between two and four (out of eight for most linkage groups). Interestingly, only the sires appeared to be segregating for colour on Chr 4, which could be explained by the lack of male recombination enabling detection in the sires only, when the underlying variation is actually located some distance away from the nearest marker. One possible weakness of the across-family half-sib analysis as undertaken here is that low QTL heterozygosity in the parents reduces the power of detection [44]. The optimal solution to the analysis of this F2-type dataset could be a combined half-sib/line-cross model, as suggested by Kim et al. [45]. The estimates of the proportion of phenotypic variance explained by the QTL under the line-cross model were substantially smaller than under the half-sib model (the largest QTL for flesh colour explained only 3.7% of the phenotypic variance in the line-cross model vs. 12.6% in the half-sib model). This is probably due to the fact that the F0 lines were outbred and therefore the estimated QTL effects were underestimated [42]. If in such a situation the data are analysed using a line-cross model, the estimated additive effect will be reduced by a fraction (p_H _- p_L_), where p_H _is the frequency in the H line and p_L _is the frequency in the L line.

Clearly, these results should be further validated with a denser marker map and additional families, since the QTL could only be mapped to relatively broad chromosomal regions. A relatively dense Atlantic salmon SNP chip, recently developed at the Centre for Integrative Genetics (CIGENE) in Norway in collaboration with international partners and containing 5000-7000 polymorphic SNP, may be a useful tool for this purpose. These SNP arrays offer much more efficient genotyping and scoring, and can be relatively inexpensive when coupled with methods such as selective DNA pooling [46,47]. The increased marker density of this SNP array will not only help close the gaps that are present in the current linkage map, but may facilitate the use of linkage disequilibrium information to further fine-map QTL.

Nevertheless, this study presents useful evidence for QTL of the important commercial and biological trait of flesh colour, and provides additional information on QTL for commercially important growth traits. There is of course a risk that QTL segregating in a resource population like that used in this study may not be found in commercial populations. However, if this should be the case, the QTL identified in the present study still contribute to a better understanding of the genetic control and biological mechanisms underlying the metabolism of dietary pigments in salmon, and the genetic architecture of growth traits in this species.

## Conclusions

A large number of significant and suggestive QTL for flesh colour and growth traits were found in an F2 cross between a landlocked and a commercial strain of Atlantic salmon. Chr 26 and Chr 4 presented the strongest evidence for significant QTL affecting flesh colour, while Chr 10, Chr 5 and Chr 4 presented the strongest evidence for significant QTL affecting growth traits (length and weight). These QTL could be strong candidates for use in marker-assisted selection and may provide further insight into the genetic control of flesh colour and growth traits in this species.

## Competing interests

The authors declare that they have no competing interests.

## Authors' contributions

DIV coordinated and supervised the study. MB performed the laboratory work with assistance from TM, conducted the data analyses and wrote the manuscript with contributions from TM and DIV. All authors read and approved the final manuscript.

## Supplementary Material

Additional file 1 **Markers used and map positions**. List of markers and female map positions used in this study. Click here for file

Additional file 2 **QTL effects for segregating parents**. Allele substitution effects and absolute t-values for segregating parents (QTL at least chromosome-wide significant at P < 0.01). Click here for file
